# Treatment of neuropathic pain in diabetic rats with synergistic combinations of nortriptyline + tramadol and nortriptyline + ibuprofen

**DOI:** 10.3389/fphar.2026.1814008

**Published:** 2026-09-08

**Authors:** M. Gicela Pérez-Hernández, Alberto Sesena-Rubfiaro, Ángel D. Trillo-Arenazas, Yolitzy Cárdenas, Miguel Huerta, Xóchitl Trujillo, Enrique A. Sánchez-Pastor, M. Irene Díaz-Reval

**Affiliations:** 1 Faculty of Nursing, University of Colima, Colima, Mexico; 2 Molecular Sciences, A.I. Virtanem Institute, University of Eastern Finland, Kuopio, Finland; 3 University Center for Biomedical Research, University of Colima, Colima, Mexico

**Keywords:** neuropathic pain, synergism, diabetes, nortriptyline, tramadol, ibuprofen

## Abstract

**Introduction:**

Neuropathic pain in diabetic patients is very difficult to treat and does not respond to conventional analgesic treatments. It has been observed that tricyclic antidepressants are able to reduce this type of pain. In some animal experimental models, drug combinations have yielded better results. Thus, this study analyzed the type of synergism produced by the combinations of Nortriptyline + Tramadol and Nortriptyline + Ibuprofen in streptozotocin-induced diabetic rats.

**Methodology:**

Hyperglycemia was induced by administering 65 mg/kg i.p. of streptozotocin to Wistar rats. The formalin model was used to evaluate hyperalgesia with administration of formalin at 0.5%. The effects of tramadol, ibuprofen, and nortriptyline as monotherapies were evaluated. The doses administered in combination were determined using the mean effective doses of each drug and the constant-ratio method. An isobolographic analysis was performed to determine the type of synergism.

**Results:**

Nortriptyline, Tramadol, and Ibuprofen showed an antihyperalgesic effect dose-dependent. Nortriptyline was the most effective and most potent; its maximum effect was 96.67% ± 1.52%, and the ED_50_ was 3.0 mg/kg. The Nortriptyline + Tramadol combination showed similar efficacy to that of Nortriptyline monotherapy. However, the ED_50_ for its combination was significantly lower (0.97 mg/kg). The isobolographic analysis showed that this combination generates supra-additive synergism, which was confirmed with the interaction index (γ = 0.237). The combination of Nortriptyline + Ibuprofeno exhibited a maxim effect of 81.36% ± 2.36%, and the ED_50_ was 15.6 mg/kg, its interaction index was less than the unit (γ = 0.699) this indicates a supra-additive synergism.

**Conclusion:**

Our study demonstrates that combinations of Nortriptyline with either Tramadol or Ibuprofen are more effective at lower doses than the drugs alone. A supra-additive synergism has been observed for both combinations, which may be useful in the management of neuropathic pain caused by diabetes.

## 1 Introduction

Neuropathic pain (NP) is estimated to affect approximately 7%–10% of the general population and is associated with a substantial reduction in quality of life. This is largely attributable to its chronic course and the limited efficacy of currently available therapeutic interventions (Baskozos et al., 2023). NP results from injury or disease of the somatosensory system (Finnerup et al., 2021). It is characterized by pathological hypersensitivity to noxious stimuli (hyperalgesia) and by nociceptive responses to non-noxious stimuli (allodynia) (Cavalli et al., 2019). Diabetes is considered one of the most common causes of NP, with chronic hyperglycemia serving as a critical pathogenic factor. High glucose levels promote the degeneration of intraepidermal nerve fibers, resulting in peripheral and central hyperexcitability (Ved et al., 2018). Patients typically present with symmetrical polyneuropathy, with symptoms that begin in the feet and progress proximally to affect the lower legs and subsequently the hands. Symptoms include intense, burning pain accompanied by allodynia, hyperalgesia, and autonomic nervous system dysfunction (Finnerup et al., 2021). Experimental animal models have provided evidence that helps us to understand some of the mechanisms underlying this disease. In the peripheral nervous system (PNS), changes in gene expression patterns and ion channel activity have been observed, leading to ectopic activity. Meanwhile, in the central nervous system (CNS), there is increased synaptic transmission and disinhibition at the spinal and supraspinal levels, leading to amplified central processing (Zuidema et al., 2024).

Clinical guidelines have been established for the management of NP. The Japanese Society of Pain Physicians recommends prioritizing agents with moderate to high efficacy, identifying tricyclic antidepressants (TCAs) and gabapentinoids as first-line treatments, selective serotonin and norepinephrine reuptake inhibitors (SNRIs) as second-line treatments, and opioid analgesics, such as tramadol, as third-line options (Nishikawa and Nomoto, 2017). Similarly, the Canadian Pain Society guidelines also designate TCAs as first-line therapy but also including include cannabinoids as third-line agents (Mu et al., 2017). This highlights the lack of consensus regarding the most effective pharmacological interventions for NP. Consequently, despite the availability of multiple therapeutic options, most patients achieve only partial relief when these drugs are administered as monotherapy.

The World Health Organization has recommended the use of the analgesic ladder as the simplest and most valuable pain management guidance, which includes combinations of analgesics with distinct mechanisms of action or combinations of analgesics and adjuvants. Evidence from several studies suggests that a synergistic effect can be achieved by combining drugs with different mechanisms of action (Chou, 2006; Díaz-Reval et al., 2022). In clinical practice, the synergistic effects for pain relief have been classified as additive, supra-additive, or sub-additive effects (Tallarida, 2012). The most desirable form of synergy is the supra-additive effect, as it not only enhances therapeutic efficacy but also allows the use of lower doses of each drug within the combination, thereby potentially reducing adverse effects.

Nortriptyline is a TCA used as a first-line treatment for neuropathic pain, as is amitriptyline, which is a prodrug; nortriptyline is its primary metabolite. The main mechanism of action of nortriptyline is the inhibition of serotonin and norepinephrine reuptake (Cavalli et al., 2019); however, it blocks the norepinephrine transporter more potently than amitriptyline. In addition, nortriptyline blocks sodium channels (Finnerup et al., 2021), which have been shown to be active in neuropathic pain. These mechanisms contribute to their effectiveness in pain relief. It has also been reported that, because it is a secondary amine, it is better tolerated than amitriptyline (Jang and Oh, 2023). Clinical studies report that it provides analgesic efficacy comparable to morphine for chronic lumbar pain, it is less effective than duloxetine in treating peripheral diabetic neuropathy (Hashemzadeh et al., 2024).

Tramadol is an analgesic classified as an atypical opioid, acting through µ-opioid receptors while also inhibiting serotonin and noradrenaline reuptake (Mu et al., 2017; Nishikawa and Nomoto, 2017; Raffa, 2001). There is evidence (Mian et al., 2024) that tramadol has demonstrated efficacy in the treatment of neuropathic pain comparable to that of antidepressants, which is attributed to its mechanisms of action; therefore, systemic administration of nortriptyline and tramadol allows them to reach the central nervous system, where these drugs modulate the control of nociceptive signaling in essential regions, such as the spinal cord.

Ibuprofen is a nonsteroidal anti-inflammatory drug (NSAID); its main mechanism of action is to inhibit the prostaglandin synthesis. This analgesic is used to treat mild to moderate pain and is commonly available over the counter. There have been no reports of its use in humans for the treatment of neuropathic pain caused by diabetes. However, studies in rodents using NSAIDs such as meloxicam or ketorolac have shown that these drugs reduced hyperalgesia and allodynia in nerve ligation models (Park et al., 2019). In experimental models of diabetes-induced neuropathy, elevated prostaglandin release has been observed in affected regions such as the spinal cord, where ibuprofen has been shown to exert an antinociceptive effect (Ortega-Álvaro et al., 2012). This suggests that nerve damage activates the COX pathway, which may play an important role in nociceptive processing, but this has not yet been fully investigated. Therefore, a treatment that combines drugs with different mechanisms of action may be promising. The aim of this study was to explore the synergistic effects of Nortriptyline + Tramadol and Nortriptyline + Ibuprofen combinations in hyperglycemic rats with neuropathic pain.

## 2 Materials and methods

### 2.1 Experimental animals

Adult male Wistar rats, weighing 230–250 g, acquired from the vivarium of the University Center for Biological Research of the University of Colima (Colima, Mexico), were used in this study. They were kept at a temperature between 22 °C and 24 °C, with 12-h light/dark cycles, standard food and water were provided free demand. All experiments were performed considering the Guidelines on Ethical Standards for Experimental Pain Research in Animals (Zimmermann, 1983), the official Mexican standard NOM-062-ZOO-1999 for care and use of laboratory animals, as well as guidelines of the Ethics Committee (approval 2019-04) and the Internal Committee for the Care and Use of Laboratory Animals of the University Center for Biomedical Research from University of Colima (approval 2021-4DD).

### 2.2 Drugs

Streptozotocin (SO130) was dissolved in ice-cold distilled water and light-protected for its immediate administration. Tramadol hydrochloride (42965-F), ibuprofen sodium salt (I1892), nortriptyline hydrochloride (N7261), and formaldehyde (F1635) were prepared in a 0.9% saline solution. All these drugs were obtained from Sigma-Aldrich. On the other hand, gabapentin (2JM281A) was purchased from PharmaLife, and was dissolved in physiologic saline solution.

### 2.3 Diabetes induction

Streptozotocin (65 mg/kg) was intraperitoneally administered to induce experimental diabetes in rats. It was dissolved in ice-cold distilled water; this solution was administered freshly to animals with 12 h fasting. Control animals underwent the same procedures but did not receive the drug treatment (Ceseña and Calcutt, 1999; Juárez-Rojop et al., 2015). Diabetes was confirmed when they maintained hyperglycemia equal to or greater than 250 mg/dL for 3 weeks. Glycemia was measured before induction, and at 7, 14, and 21 days after. If at 21 days glucose was equal to or greater than 250 mg/dL, the rats were considered to have developed hyperglycemia. Blood glucose levels were monitored using an Accu-Check Performa glucometer. Following the methods established in previous studies (Dominguez et al., 2015), the blood for glucose measurements was obtained via a tail-tip snip after a 12-h fasting period. This ensured sufficient blood was available for the test strip and confirmed hyperglycemia for at least 3 weeks before the experiments commenced.

### 2.4 Evaluation of hyperalgesia

The formalin test (0.5% concentration) was used to assess hyperalgesia. This model involves subcutaneously injecting 50 µL of the formalin solution (0.5% or 2%) into the dorsal surface on the rat’s hind leg (Wheeler-Aceto and Cowan, 1991). Following a 30 min acclimation period in a Plexiglas chamber, the formalin was injected, and the animals were returned to the chamber for observation. Nociceptive behavior, specifically paw flinching, was recorded at 5-min intervals for 1 h. The formalin test exhibits a biphasic response as described previously (Díaz-Reval et al., 2010; Wheeler-Aceto and Cowan, 1991). Under our experimental conditions, phase 1 occurred during the first 10 min, followed by phase 2, which lasted from 15 to 60 min. To ensure ethical treatment, each animal was used only once and humanely euthanized by cervical dislocation at the end of the experiment.

### 2.5 Experimental design

The following experimental groups were used: three groups of rats were used to determine the induction period for neuropathic pain under our experimental conditions. Two of these groups were administered ice-cold distilled water intraperitoneally (i.p.) and were identified as healthy rats (HR). Hyperalgesia was assessed after a 21-day period; one group was treated with 2% formalin and the other with 0.5% formalin. The third group was administered 65 mg/kg (i.p.) streptozotocin (STZ) and was identified as hyperglycemic rats (DR); in these rats, hyperalgesia was assessed by applying 0.5% formalin. All rats included in the study were weighed and had their glucose levels measured weekly for 21 days.

In this study, the isobolographic method was used to determine the type of synergy (Tallarida, 2012); therefore, to determine the dose-response curves (DRCs) of the drugs all test groups were administered as follows the hyperglycemic control group received only saline solution subcutaneously (s.c.) and intraperitoneally. Other group was administered with gabapentin at dose of 50 mg/kg s.c. 30 min before formalin; this group was used as positive control. The experimental groups were administered with Tramadol at doses of 2.5, 5, 10, and 20 mg/kg s.c. Another group received Nortriptyline (i.p.) at doses of 1, 3, 10, and 30 mg/kg. The Ibuprofen group was administered with 10, 40, 80, and 160 mg/kg s.c. Tramadol and Ibuprofen were administered 15 min before formalin injection. The doses were selected based on previous studies conducted in our laboratory (Díaz-Reval et al., 2010; Díaz-Reval et al., 2022). Nortriptyline was administered 25 min before formalin injection, and the doses were selected based on the study conducted by Miranda et al. (2015). Logarithmically increasing doses were used for all DRCs.

With the results of previous experiments, DRC for each drug were built, and then the median effective dose (ED50) was calculated. The constant ratio method was used to calculate the doses of each drug administered in combination, with the drugs given at a predetermined, constant ratio throughout the experiment. This method allows us to compare the observed antihyperalgesic effect of the drug combination at each dose level. Six doses of Nortriptyline (0.093, 0.188, 0.375, 0.75, 1.5, and 3 mg/kg, i.p.) were paired with corresponding doses of Tramadol (0.162, 0.325, 0.650, 1.30, 2.60, and 5.20 mg/kg, s.c.). This yielded six combination doses (0.255, 0.512, 1.025, 2.05, 4.10, and 8.20 mg/kg). On the other hand, four doses of Nortriptyline (0.375, 0.75, 1.50, and 3 mg/kg, i.p.) were administered in combination with corresponding doses of Ibuprofen (5.2, 10.4, 20.8, and 41.6 mg/kg, s.c.). Subsequently, this resulted in total doses of 5.575, 11.15, 22.30, and 44.60 mg/kg.

The animals that received the treatments described above were administered with streptozotocin 3 weeks before performing the experiments with 0.5% formalin, if they showed hyperglycemia equal to or greater than 250 mg/dL (DR). Each dose was tested with n = 6.

### 2.6 Data presentation and statistical analysis

In accordance with ethical principles for the study of pain in animals, the sample size was calculated using the resource equation (Charan and Kantharia, 2013; Arifin and Zahiruddin, 2017), which incorporates the principles of the 3Rs (Reduction, Refinement, and Replacement). This calculation was performed to determine the minimum sample size required to detect the probability of an error in an analysis of variance. The equation takes into account.

Minimum number of animals per group (mnapg)

$$mnapg=\left(\frac{10}{K}\right)+1$$

Maximum number of animals per group (MNAPG)

$$MNAPG=\left(\frac{20}{K}\right)+1$$

Where K is the number of treatments, 10 and 20 are the minimum and maximum degrees of freedom, respectively, for a one-way ANOVA. The experimental design describes each treatment, which involves different doses; the calculation was performed individually for each treatment, taking the doses into account, and then standardized for all treatments.

Nociceptive behavior data were used to calculate antihyperalgesic effects. The area under the curve (AUC) for each time course was computed using the trapezoidal method. This AUC value reflects the total hyperalgesic behavior over time. Finally, the antihyperalgesic effect was determined as per cent of maximum possible effect (%MPE), according to the following formula:

$$\%\;MPE=\left(\frac{{AUC}_{C}-{AUC}_{D}}{{AUC}_{C}}\right)100$$

Where AUC_C_ is calculated from the control group, and AUC_D_ is calculated from the group administered the drug.

The isobolographic method was used to determine the type of synergism (Tallarida, 2001). The ED_50_ values for each drug administered as monotherapy were calculated and plotted on a graph, joining them with a straight line that represents the line of additivity with a 95% confidence limit. The theoretical ED_50_ (Theo) was located on this line. On the other hand, the antihyperalgesic effect of the combinations was determined experimentally, and their ED_50_ was determined, which is identified as experimental ED_50_ (Exp), and subsequently placed on the isobologram. If the Exp ED_50_ is located below the line of additivity, the combination exhibits supra-additive synergism; if it falls on the line, it exhibits additive synergy; finally, when the Exp ED_50_ is located above the additivity line, the combination shows infra-additive synergism. This is verified by the interaction index (γ), because the method predicts supra-additive synergism when γ < 1.

All data were expressed as the mean ± standard error of the mean (S.E.M.) for n = 6 for each treatment. An analysis of variance (ANOVA) was used for multiple comparisons, followed by the *post hoc* Tukey test. Statistical significance was defined as p < 0.05. Statistical analysis, the determination of ED_50_, and creation of graphics were performed with GraphPad Prism 10.3.0 (GraphPad Software, San Diego, CA, United States).

## 3 Results

### 3.1 Physiological markers

**FIGURE 1:**
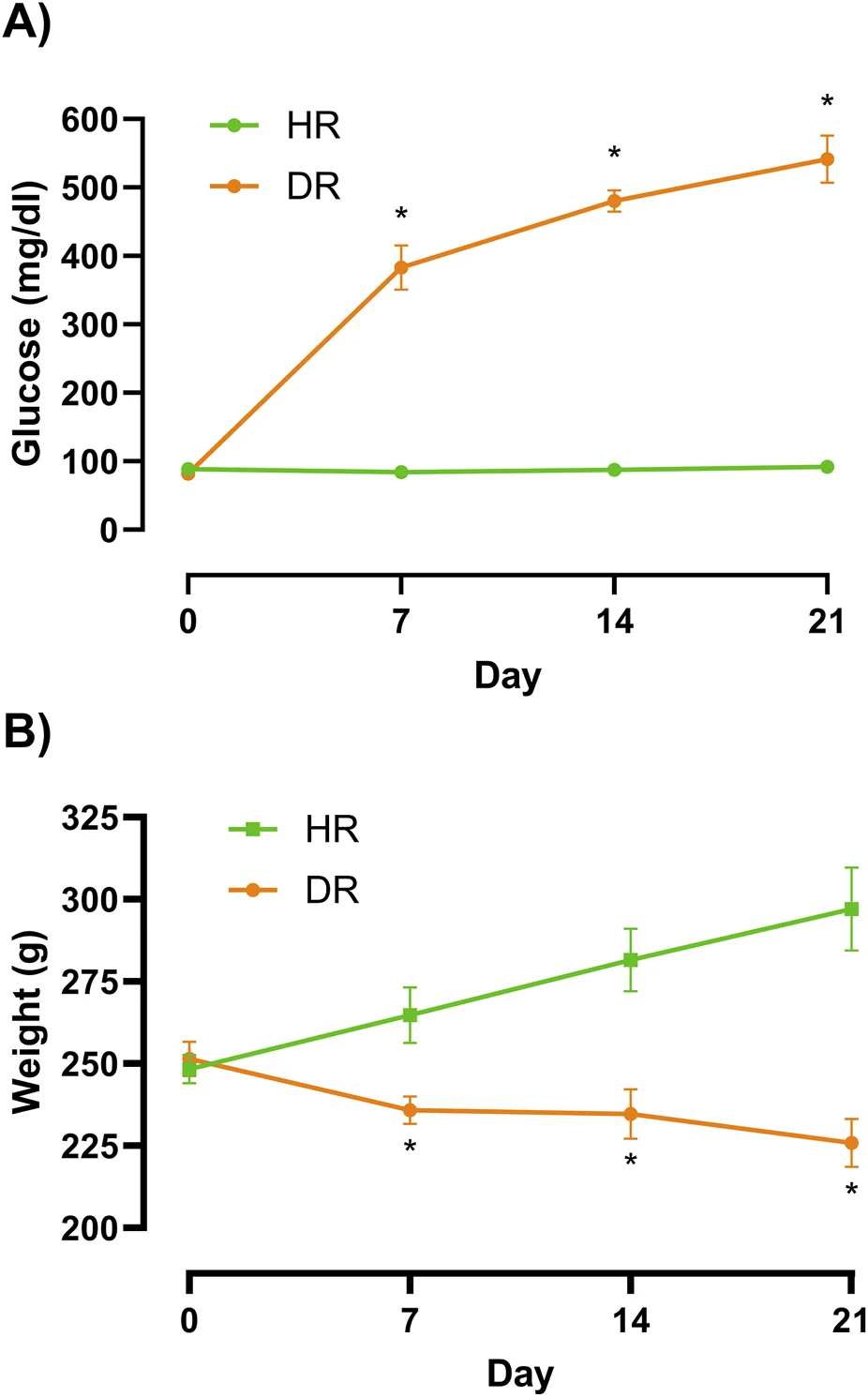
Physiological markers after administration of STZ (65 mg/kg). **(A)** shows glucose levels in healthy rats (HR) and diabetic rats (DR), evaluated over 21 days. The increase in glucose in DR rats was significant compared to HR rats (p < 0.0001). **(B)** shows the change in body weight in both groups of animals. There was a statistically significant difference between the two groups (p = 0.0021). Each point represents the average of n = 6 ± SEM.

Two groups of rats, HR rats and STZ-induced DR rats were used to determine the time course of neuropathic pain development. Blood glucose levels and body weight were monitored weekly for 21 days. As expected, the DR group developed chronic diabetes, characterized by hyperglycemia compared to the HR rats (Figure 1A). Following STZ injection, blood glucose levels increased significantly throughout the 21-day period (p < 0.0001). Conversely, HR rats maintained basal blood glucose levels during the same period. Blood glucose level alterations were accompanied by changes in body weight. By day 21, DR rats exhibited a decrease in body weight, while HR rats displayed a significant increase (p = 0.0021) in body weight (Figure 1B). Additional diabetic symptoms observed in DR rats included polyphagia, polyuria with glycosuria, and decreased motor activity compared to HR rats (data not shown). The test groups were treated identically to the DR group. Body weight and blood glucose levels were comparable to those presented in Figure 1. On day 21, immediately after blood glucose measurement, the rats were weighed and treated with the study drugs, either as monotherapy or in combination, according to the experimental protocol. They were then subjected to the formalin test.

### 3.2 Evaluation of hyperalgesia

**FIGURE 2:**
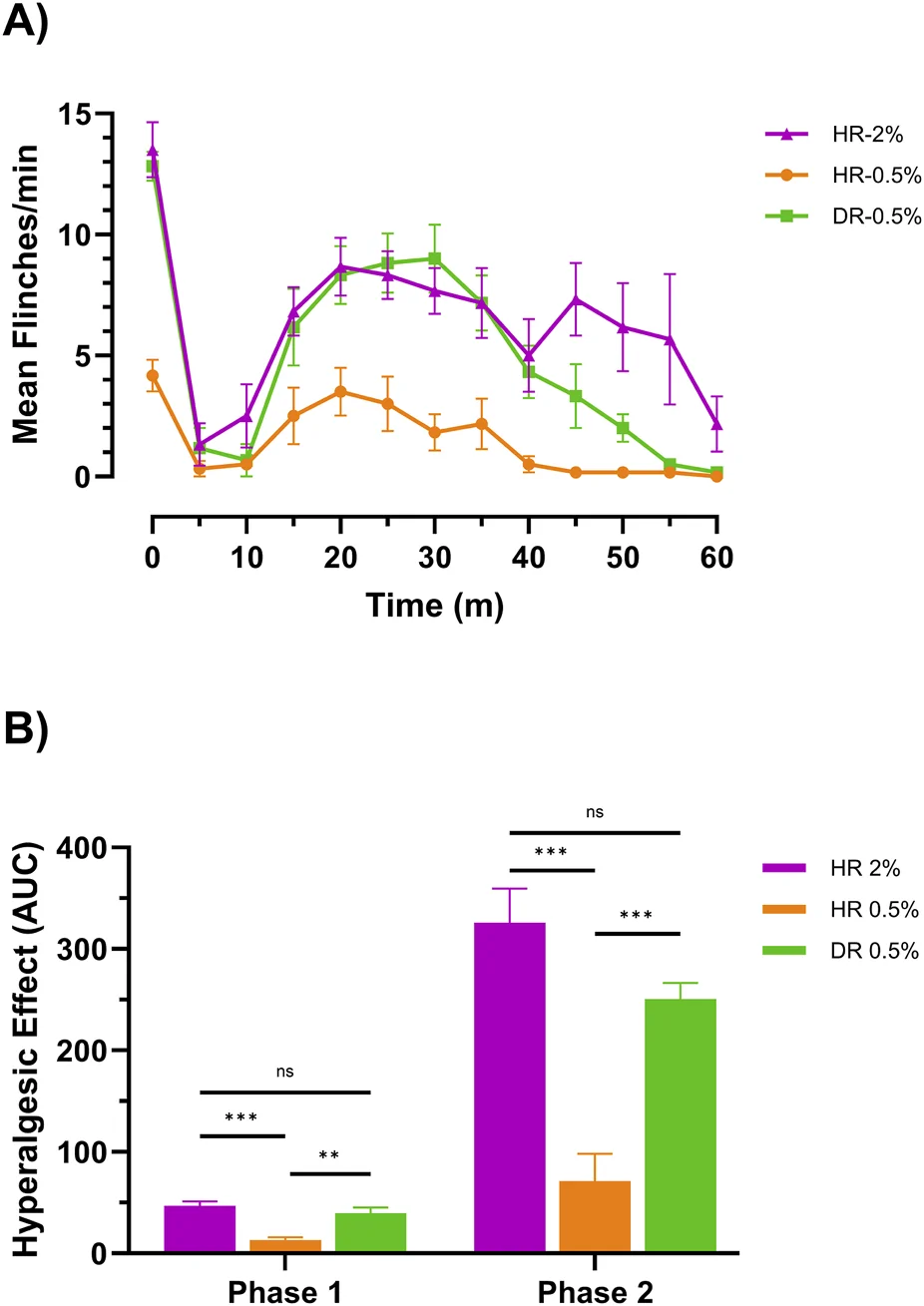
Hyperalgesic effect in rats treated with STZ. **(A)** shows the behavior over time of rats administered formalin 21 days after STZ administration. HR-2% was the group of healthy rats administered with 2% formalin, HR-0.5% represents the group of healthy rats administered with 0.5% formalin, and DR-0.5% indicates the group of diabetic rats treated with 0.5% formalin. **(B)** shows the overall hyperalgesic effect evaluated as the area under the curve (AUC) for both phases of the formalin model. Each group consisted of n = 6. ** (p < 0.01), *** (p < 0.001), ns indicates not significant.

We compared the nociceptive behavior by employing the intraplantar formalin in HR and DR rat groups. The temporal courses show similar behavior in phase I and up to minute 40 of phase II between the HR group administered with 2% formalin and the DR group administered with 0.5% formalin. While another group of healthy rats that received 0.5% formalin showed a substantially lower nociceptive effect in both phases of the formalin test (Figure 2A).

Upon administration of formalin at 0.5%, DR rats exhibited a significantly higher average number of flinches (p < 0.001) compared to HR rats in both phase 1 and phase 2 of the model (Figure 2B). The cumulative nociceptive effect, calculated as the area under the curve (AUC) for HR rats during phase 1 of the formalin test at 0.5% and 2%, was 13.3 ± 3 and 46.7 ± 4, respectively. In contrast, for DR rats, administered with formalin at 0.5% was 29.1 ± 8. During phase 2, the AUC for HR rats that received formalin at 0.5% and 2% was 71.3 ± 27 and 325.8 ± 34, respectively. While for DR rats administered with formalin at 0.5%, the AUC was 285 ± 64. Interestingly, administering formalin at 2% in HR rats produced similar results to formalin at 0.5% in DR rats. These findings show the presence of hyperalgesia, an increased sensitivity to pain stimuli, in DR rats compared to HR controls, at 21 days after administration of streptozotocin.

### 3.3 Evaluation of the synergism of the combination of nortriptyline + tramadol

Following confirmation of neuropathic pain at 21 days post-hyperglycemia induction, we assessed the antihyperalgesic effects of Nortriptyline, Tramadol, and Ibuprofen in the formalin model. Previous studies have demonstrated that several anti-inflammatory analgesics, including NSAIDs such as Ibuprofen, do not affect the first phase of the formalin model. Consistent with these observations, Nortriptyline also failed to modulate nociceptive responses in this phase under our experimental conditions (data not shown). Therefore, the findings presented here correspond exclusively to the second phase of the formalin model.

**FIGURE 3:**
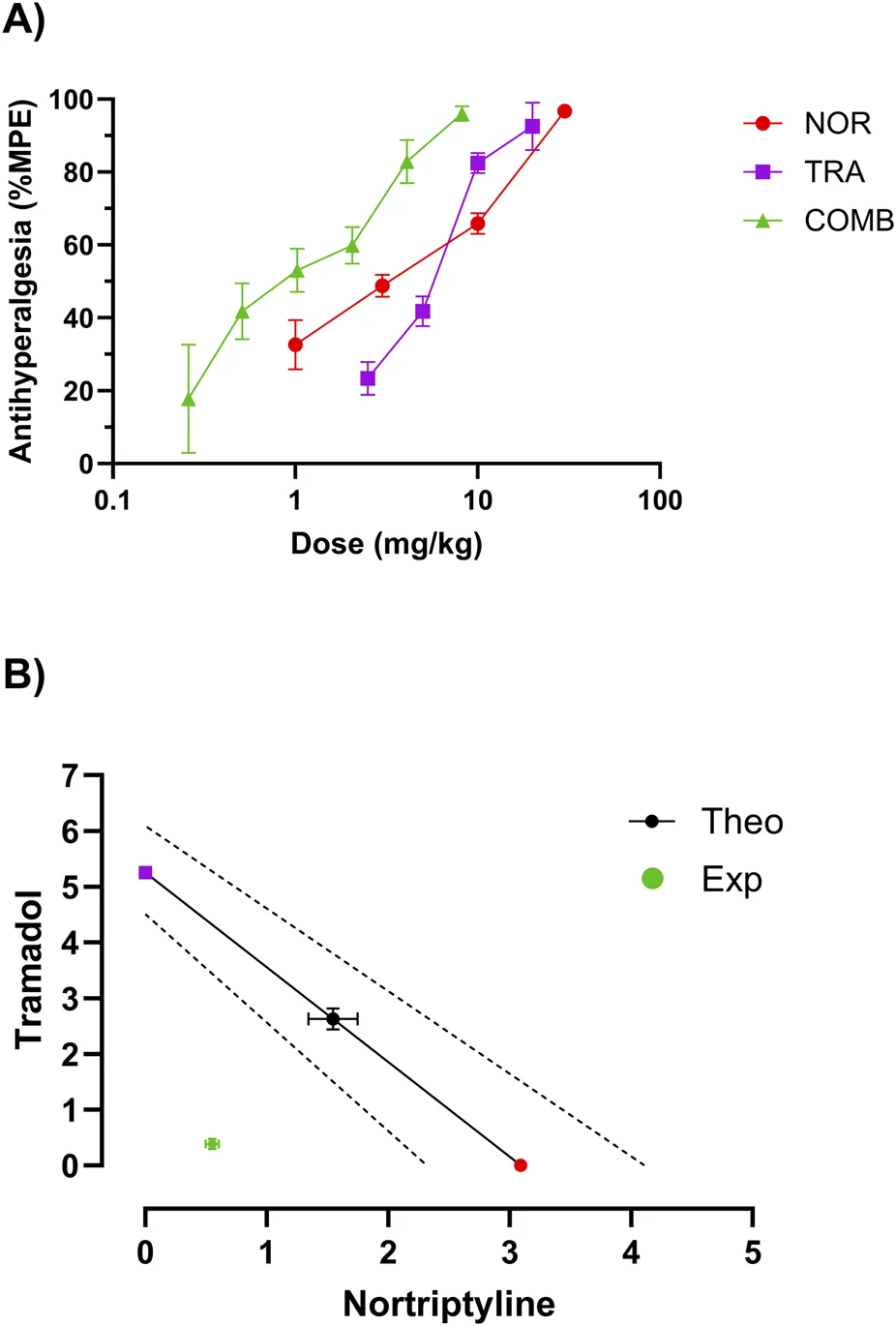
Antihyperalgesic synergism of the combination of Nortriptyline and Tramadol. **(A)** shows the DRCs for monotherapy with Nortriptyline (NOR), Tramadol (TRA), and their combination (COMB). **(B)** shows the isobologram for the combination. The X-axis shows the ED_50_ of Nortriptyline, and the Y-axis shows the ED_50_ of Tramadol. The solid line represents the isobola on which the theoretical ED_50_ (Theo) of the combination is located. The experimental ED_50_ (Exp) is below the isobola. The dotted lines indicate the confidence limit of the additivity region. Each dose was evaluated with n = 6.

As shown in Figure 3A, the Tramadol DRC exhibits a clear dose-dependent effect, starting at 2.5 mg/kg, that resulted in antihyperalgesic response of 23.4% ± 4.53%. The increase in the effect between 5 and 10 mg/kg is particularly notable, rising from 41.82% ± 4.15% to 82.58% ± 2.74%, and reaching a maximal antihyperalgesic efficacy of 92.58% ± 6.51% at 20 mg/kg. In contrast, the lowest Nortriptyline dose evaluated (1 mg/kg) produced an effect of 32.61% ± 6.77%, and its DRC also demonstrated a robust dose-dependent profile, achieving a maximal efficacy of 96.67% ± 1.52%. Under these experimental conditions, both drugs, when administered as monotherapy, exhibited similar maximal efficacy. The media effective dose (ED_50_) was 3 mg/kg for Nortriptyline and 5.2 mg/kg for Tramadol (Table 1), indicating greater potency of nortriptyline at the ED_50_ level. However, due to tramadol’s curve displays a steeper slope, it becomes more potent than nortriptyline once its effect exceeds 60%.

**TABLE 1:** ED_50_ of drugs administered as monotherapy and in combination.

Drugs	ED_50_ (mg/kg)	Route
Nortriptyline	3.0	i.p.
Tramadol	5.2	s.c.
Ibuprofen	41.6	s.c.
NOR + TRA	0.97	i.p. + s.c.
NOR + IBU	15.6	i.p. + s.c.

After determining the ED_50_ values for each drug, the corresponding combination doses were selected (data shown in the methodology) to generate the dose response curve. Both drugs were administered by the same route used in monotherapy, Tramadol subcutaneously (s.c.) and Nortriptyline intraperitoneally (i.p.), at a dose range of 0.255–8.2 mg/kg.

When administered in combination, the resulting DRC shows a dose-dependent effect, which is closer to the Y-axis than the DRCs of the drugs administered alone, indicating that lower doses were required to achieve comparable effects. The maximum efficacy achieved with the combination was 96.01% ± 2.06%. There is no significant difference compared to the efficacy observed with Tramadol and Nortriptyline administered alone. However, it is important to highlight that the doses necessary to elicit this effect were clearly reduced: the effective dose of Tramadol (5.2 mg/kg) decreased 4-fold, whereas that of Nortriptyline (3 mg/kg) was reduced 10-fold.

An isobolographic analysis was conducted to evaluate the interaction between Nortriptyline and Tramadol. In this analysis (Figure 3B), the individual ED_50_ values of Nortriptyline (i.p.) and Tramadol (s.c.) were plotted on the X and Y-axes, respectively. The line connecting these points represents the isobole, indicating all theoretical dose combinations that produce an additive effect. The theoretical ED_50_ for the combined drugs was 4.1 mg/kg.

The experimental results demonstrated a synergistic effect. The actual ED_50_ obtained for the combination (0.97 mg/kg) was significantly lower (p < 0.05) than the theoretical ED_50_ and located below the isobole curve. This difference was further confirmed by the interaction index (γ = 0.237), supporting a supra-additive synergism. Demonstrating, that combined effect of Nortriptyline and Tramadol exceeded the sum of their individual effects.

### 3.4 Evaluation of the synergism of the combination of nortriptyline + ibuprofen

**FIGURE 4:**
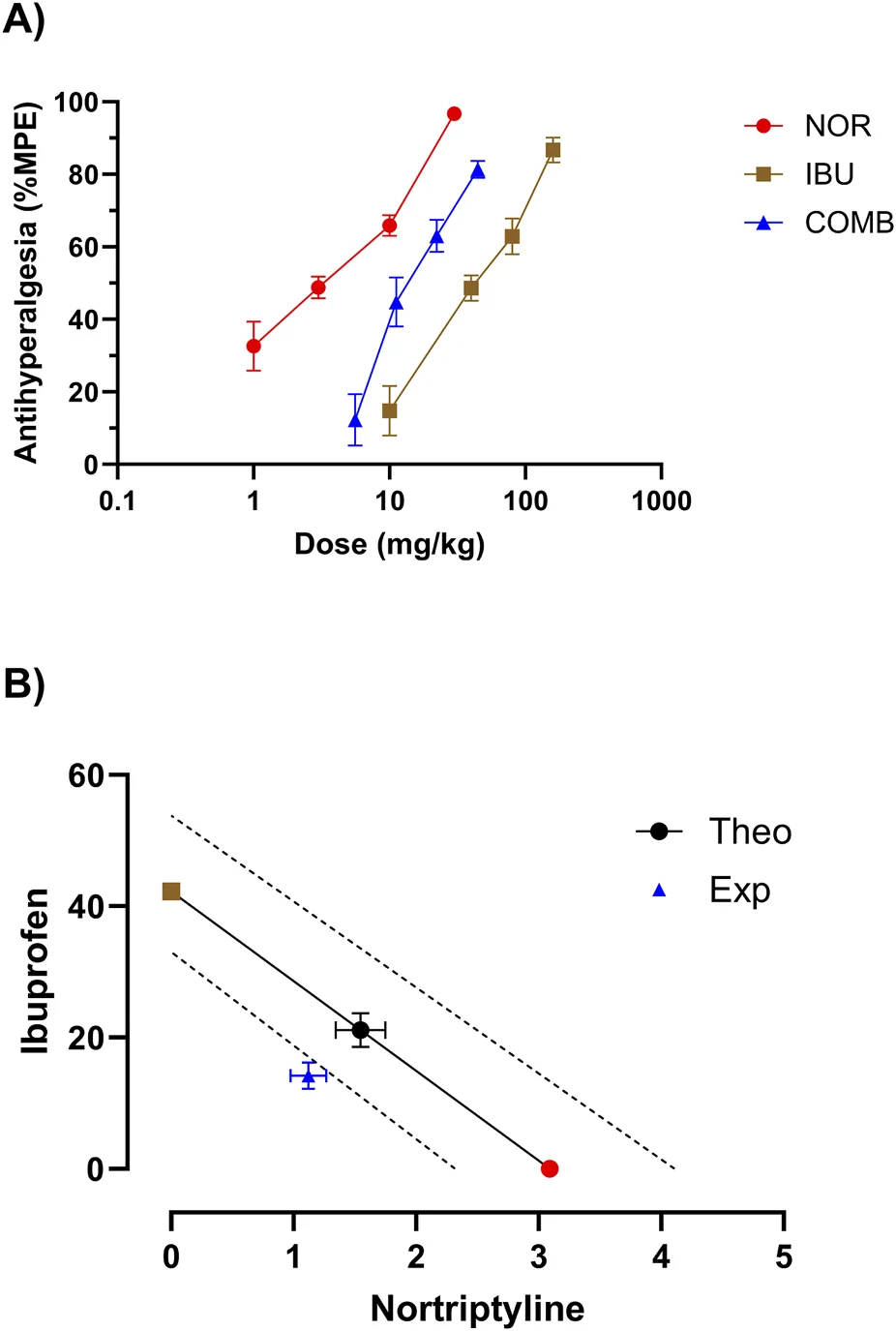
Antihyperalgesic synergism of the combination of Nortriptyline and Ibuprofen. **(A)** shows the DRCs for monotherapy with Nortriptyline (NOR), Ibuprofen (IBU), and their combination (COMB). **(B)** shows the isobologram for the combination. The X-axis shows the ED_50_ of Nortriptyline, and the Y-axis shows the ED_50_ of Ibuprofen. The solid line represents the isobola on which the theoretical ED_50_ (Theo) of the combination is located. The experimental ED_50_ (Exp) is below the isobola. The dotted lines indicate the confidence limit of the additivity region. Each dose was evaluated with n = 6.

Furthermore, this study also assessed the potential synergistic interaction between Nortriptyline and Ibuprofen. Figure 4A presents the DRC for each drug administered as monotherapy and in combination. The DRC for Ibuprofen began at 10 mg/kg, producing an antihyperalgesic effect of 14.81% ± 6.83%, and exhibited a clear dose-dependent profile, reaching a maximal effect of 86.69% ± 3.41% under these experimental conditions. As noted previously, nortriptyline produced the greatest efficacy in this study at a dose of 30 mg/kg.

When both drugs were co-administered, the DRC started at a total dose of 5.58 mg/kg (12.31% ± 7.14%), and the maximal effect reached was 81.36% ± 2.36%, comparable to that obtained with Ibuprofen alone. However, achieving this effect with combination therapy required a substantially lower total dose (44.6 mg/kg) than monotherapy. Notably, the Ibuprofen dose was reduced 4-fold, while the Nortriptyline dose was reduced 10-fold. As illustrated in Figure 4A, the DRC for the combination falls between the DRC of the individual drugs, indicating a potency greater than ibuprofen but lower than nortriptyline, consistent with the mean effective doses reported in Table 1.

The isobologram shown in Figure 4B further supports synergistic antihyperalgesic interaction in hyperglycemic rats. The experimentally determined ED_50_ (15.6 mg/kg) was lower than the theoretical ED_50_ (22.3 mg/kg), placing it below the line of additivity. Statistical analysis confirmed a significant difference between these values (p < 0.05). Together, these findings demonstrate that the Nortriptyline + Ibuprofen combination produces a synergistic effect, corroborated by an interaction index of γ = 0.699.

### 3.5 Maximum efficacy of drugs combination

**FIGURE 5:**
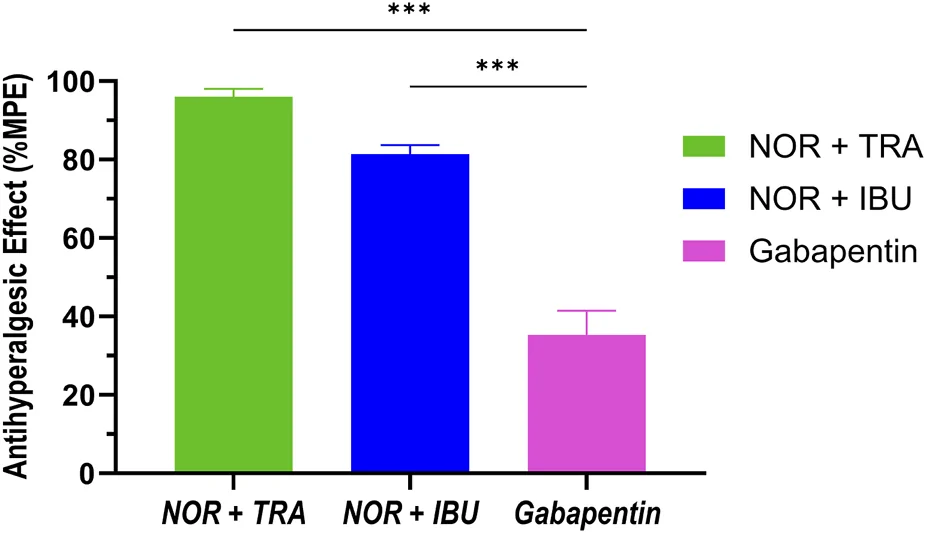
Maximum antihyperalgesic effect of combinations. The maximum effect of both the combination of Nortriptyline + Tramadol (NOR + TRA) and the combination of Nortriptyline + Ibuprofen (NOR + IBU) is compared with Gabapentin, used as a positive control in this study. *** (p < 0.0001), the mean of n = 6 ± SEM is plotted.

The comparison of maximum efficacy (Emax) between the drug combinations and Gabapentin, used as the positive control group, is illustrated in Figure 5. Gabapentin, a well-established antiepileptic drug for neuropathic pain, was administered subcutaneously at a dose of 50 mg/kg, 30 min prior to induction of the hyperalgesic state with formalin at 0.5%. Notably, Gabapentin demonstrated a lower efficacy of 35.27% at 50 mg/kg. In contrast, the combination of Nortriptyline + Tramadol showed a significantly higher efficacy of 96.01% at 8.2 mg/kg (p = 0.0001), and the Nortriptyline + Ibuprofen combination displayed an efficacy of 81.36% at 44.6 mg/kg (p = 0.0001). Under these experimental conditions, the combinations are more effective than the first-line drug used in clinical practice.

## 4 Discussion

### 4.1 Hyperalgesic effect

In this study, we analysed the synergic effect of Nortriptyline + Tramadol and Nortriptyline + Ibuprofen in the STZ-induced diabetic rat model. Our findings on hyperglycemia induction are consistent with previous studies (Furman, 2021; Juárez-Rojop et al., 2015; Wang-Fischer and Garyantes, 2018) showing significant increases in blood glucose levels compared to the healthy rat. Additionally, the observed weight loss in STZ-treated rats compared to controls over 3 weeks aligns with previous reports (Fox et al., 1999; Ma et al., 2015). This weight loss, a known consequence of hyperglycemia in humans and rodents, leads to the breakdown of stored triglycerides and the release of significant quantities of fatty acids into the bloodstream, culminating in diabetic ketoacidosis. This condition presents with gastrointestinal disturbances, leading to electrolyte loss and dehydration (El-Remessy, 2022; Sztalryd and Kraemer, 1995).

The formalin test, a well-validated method for assessing nociception, revealed hyperalgesia in our experimental conditions, in accordance with findings from previous studies (Freshwater and Calcutt, 2005; Isaac Rocha-González et al., 2014). Formalin injection triggers localized tissue damage and inflammation, which is known to be exacerbated in hyperglycemia (Breitinger and Breitinger, 2023; Ronchetti et al., 2017). This enhanced inflammation contributes to the observed hyperalgesia. Additionally, hyperglycemia can alter the function of pain receptors, such as TRPV1, making them more responsive to painful stimuli like formalin (Bishnoi et al., 2011; Callaghan et al., 2012). These findings support the presence of neuropathic pain in our experimental conditions and reinforces the foundation for further investigation of the antihyperalgesic effects of the drugs tested in this study.

### 4.2 Analgesic effects of nortriptyline, tramadol, and ibuprofen in neuropathic pain

Nortriptyline, a tricyclic antidepressant, extends its therapeutic reach beyond depression management, showing promise in alleviating neuropathic pain. Preclinical studies, including our own, demonstrate its efficacy alongside other antidepressants like amitriptyline in reducing pain in rodent models (Choucair-Jaafar et al., 2014; Gilron et al., 2015; Ulugol et al., 2002). Our research, conducted on hyperglycemic rats, confirms a significant reduction in hyperalgesia following nortriptyline treatment. Mechanistically, nortriptyline inhibits the recapture of norepinephrine, increases its levels in the synaptic cleft, promote interacting with spinal α2 adrenergic receptors to inhibit calcium channels, and provides analgesia (Obata, 2017). Additionally, its analgesic effects may involve sodium channel blockade and NMDA receptor inhibition (Ozmen et al., 2020).

On the other hand, Tramadol, despite its efficacy in modulating pain perception via opioid receptor binding, is often relegated to third-line treatment for neuropathic pain due to addiction concerns (Wrzosek A et al., 2009). Our investigation on the antihyperalgesic effects of tramadol is consistent with previous studies despite differing experimental conditions, indicating dose-dependent antinociceptive effects (Caspani et al., 2014; Gong et al., 2011; Kaneko et al., 2014). While tramadol primarily acts via μ-opioid receptor binding, it also inhibits serotonin and norepinephrine reuptake, potentially increasing its analgesic properties (Grond and Sablotzki, 2004). Although its impact on sodium channels is minimal (Bok et al., 2023), emerging research suggests an indirect influence, enhancing its efficacy in neuropathic pain management.

On the other hand, our study on antihyperalgesic effects of ibuprofen in hyperglucemic rats corroborates previous findings, showing a reduction in nociceptive responses (Redondo-Castro and Navarro, 2014). Ibuprofen, besides COX inhibition, modulates sodium channel activation, and reduces inflammatory mediators, indicating its potential in treating neuropathic pain due to decreased inflammatory conditions (Amir et al., 2006; Gould et al., 2004).

### 4.3 Antihyperalgesic synergism of nortriptyline + tramadol combination

To characterize the antihyperalgesic effect of the Nortriptyline + Tramadol combination, only the effect obtained in phase two of the formalin test was considered. Isobolographic analysis revealed the type of interaction present in the Nortriptyline + Tramadol combination under our experimental conditions. The experimental ED_50_ of 0.97 mg/kg differs considerably from the theoretical ED_50_ of 4.1 mg/kg, and the interaction index is statistically less than one. Importantly, the combination achieved a maximum efficacy (96.01%) comparable to Tramadol (92.58%) alone.

Therefore, it is confirmed that the interaction is of the supra-additive type. Notably, the combination doses were significantly lower than those required for individual drugs. This showed that a lower combined dosage was sufficient to produce an equivalent antihyperalgesic effect to an individual drug, as illustrated in Figure 3. These results suggest that because the two drugs in the combination act through different mechanisms, they provide multimodal coverage for a broad spectrum of pain.

Preclinical and clinical studies have explored the interaction among various antidepressant and analgesic drugs for managing neuropathic pain. In a rat model of neuropathic pain, the interaction between tramadol and either doxepin or venlafaxine was assessed. It was observed that both drugs, whether administered individually or in combination, mitigated thermal hyperalgesia and mechanical allodynia. Notably, doxepin exhibited greater efficacy compared to venlafaxine. Moreover, the combination of tramadol and doxepin demonstrated a synergistic effect in alleviating thermal hyperalgesia, while the combination with venlafaxine showed an additive effect (Wrzosek et al., 2009).

Among tricyclic antidepressants, amitriptyline emerges as a reference molecule in preclinical studies due to its wide clinical use in psychiatry and in the treatment of pain. Although amitriptyline exhibits a greater potency (7 times) for the serotonin transporter (SERT) than for the norepinephrine transporter (NET), its active metabolite, nortriptyline, is considerably more potent in NET inhibition than in SERT, with a difference of 40 times. This suggests that a large part of the analgesic effects of amitriptyline in neuropathic pain may be attributed to its metabolite nortriptyline (Kremer et al., 2016). The greater increase in norepinephrine than serotonin in the synaptic cleft is probably the reason why Nortriptyline is effective in the treatment of neuropathic pain. It also inhibits histamine, 5-hydroxytryptamine, and acetylcholine activity, and finally blocks sodium channels (Obata, 2017; Ozmen et al., 2020). In summary, the synergistic effect observed can be explained by the different mechanisms of action of the two drugs. Nortriptyline predominantly inhibits norepinephrine reuptake, while tramadol acts as an opioid agonist and serotonin and norepinephrine reuptake inhibitor.

### 4.4 Antihyperalgesic synergism of nortriptyline + ibuprofen combination

There is limited evidence in animal models describing the analgesic effect and interaction of NSAIDs with other drug classes under nerve injury conditions. In a study, using a model of mechanical and cold allodynia induced by chronic constriction injury in rats, the effect of ibuprofen arginate versus ibuprofen sodium was investigated. Ibuprofen arginate demonstrated greater antinociceptive efficacy across various tests, including mechanical and cold allodynia, as well as in phase 2 of the formalin test (Ortega-Álvaro et al., 2012).

On the other hand, a study examined the antiallodynic impact of the combination of ibuprofen with L-arginine (a vasodilator) in rats with streptozotocin-induced diabetic neuropathy. Tactile allodynia was evaluated using Von Frey filaments. The results demonstrated that ibuprofen, L-arginine, and their combination significantly reduced tactile allodynia in diabetic rats compared to the control group (El-Lithy et al., 2016). Furthermore, an investigation into the effects of duloxetine and ibuprofen was conducted using an animal model of pain, assessed through the hot plate test and inflammatory activity induced by carrageenan administration in the rat paw. Ibuprofen notably reduced paw edema compared to both the control group and duloxetine. However, duloxetine exhibited superior analgesic effects compared to ibuprofen (Dhawale et al., 2019).

Previous studies suggest that effective therapeutic strategies for neuropathic pain management focus on the synergy derived from pharmacological mechanisms that act on different molecular targets. As observed in our results in Figure 4, isobolographic analysis was implemented to determine the drug interaction of the Nortriptyline and Ibuprofen combination under the applied experimental conditions. The experimental ED_50_, which was 15.6 mg/kg, showed a significant difference compared to the theoretical ED_50_ (22.3 mg/kg). Thus, the interaction index obtained was less than one, indicating that the interaction between both drugs is supra-additive.

The antihyperalgesic effect observed with the combination may be primarily due to nortriptyline-induced inhibition of norepinephrine reuptake, which results in an increase in its concentration in the synapse and, therefore, in an improvement in pain modulation. In addition, its inhibition of sodium channels contributes to the reduction of neuronal excitability. This variety of mechanisms underscores the utility of Nortriptyline as an effective agent in the management of neuropathic pain, offering a therapeutic option with a broad and diversified action profile.

Like many NSAIDs, ibuprofen inhibits both cyclooxygenase-1 (COX-1), which is responsible for the production of prostanoids (PG and thromboxane A_2_) that control a variety of physiological functions (vascular, blood flow, gastric, and renal functions), and COX-2, the synthesis of which increases, leading to an amplified production of PGE_2_ in inflammation and pain (Rainsford, 2009).

Recent studies have highlighted that the analgesic effects of ibuprofen are enhanced through the elevation of anandamide levels, an endocannabinoid that activates cannabinoid CB1 and CB2 receptors, thereby promoting analgesia. Ibuprofen, at therapeutic concentrations, inhibits the metabolism of anandamide, thereby augmenting its antinociceptive effect in cases of inflammatory pain. Moreover, other experiments have shown that ibuprofen can compete with synthetic agonists for binding to CB2 receptors, suggesting a mechanism through which it enhances the analgesic pathway (Boccella et al., 2023; Mazaleuskaya et al., 2015). Therefore, the synergism observed with the combination of nortriptyline and ibuprofen can be explained by the inflammatory component that exists due to nerve damage caused by hyperglycemia, a mechanism that can be inhibited by ibuprofen, and the additional mechanisms that this analgesic has to inhibit pain, in addition to the mechanisms involved in the inhibition of nociceptive signal transmission induced by nortriptyline at central level.

In this study, we compared combinations of Nortriptyline + Tramadol and Nortriptyline + Ibuprofen, which showed maximum efficacy with gabapentin, a first-line drug used in clinical practice. The dose of gabapentin administered (50 mg/kg) was similar to that used in other studies (De la O-Arciniega et al., 2009). Under these experimental conditions, gabapentin showed lower efficacy than the aforementioned combinations. The usefulness of gabapentin for the treatment of neuropathic pain is attributed to binding with high affinity to the α2δ-1 subunit of voltage-gated calcium channels (Chincholkar, 2018), which reduces calcium influx into neurons, which in turn decreases the release of excitatory neurotransmitters such as glutamate, norepinephrine, substance P, and CGRP, resulting in decreased neuronal excitability of the dorsal horn (Kukkar et al., 2013). Gabapentin has been shown to not only suppress the release of substance P but also to decrease substance P-induced activation of the NF-kB protein complex, which is an essential mediator of cytokine synthesis. Therefore, gabapentin regulates inflammation-related intracellular signaling in neuronal and glial cells, which is effective in relieving symptoms of inflammatory and neuropathic pain (Park et al., 2008). Our results demonstrated that the combinations of Nortriptyline with Tramadol, as well as Nortriptyline with Ibuprofen, were superior in efficacy to conventional treatment with Gabapentin, considered the positive control.

The results presented here show that sub-effective doses of nortriptyline and tramadol administered in combination result in greater efficacy in inhibiting hyperalgesia, a sign of neuropathic pain generated by hyperglycemia in rats, due to the inhibition of different pain mechanisms at the central level. On the other hand, the combination of nortriptyline with ibuprofen may also produce greater efficacy because, in addition to modulate pain at the central level, it interferes with inflammatory processes that may be activated by nerve damage caused by hyperglycemia. The limitations of this study are that the mechanisms of action were not analyzed, so further studies are needed to understand exactly how each of the drugs acts on neuropathic pain caused by hyperglycemia.

## 5 Conclusion

In conclusion, this study demonstrates that the combinations of Nortriptyline + Tramadol and Nortriptyline + Ibuprofen produce a supra-additive synergism, which may make them useful in the clinical practice. Furthermore, they were superior in efficacy to conventional treatment with Gabapentin, considered a first-line medication in clinical practice. These results emphasize the importance of investigating pharmacological synergism as a promising approach to developing combined therapeutic strategies that effectively address the complexity of neuropathic pain treatment.

## Data Availability

The original contributions presented in the study are included in the article, further inquiries can be directed to the corresponding author.

## In memoriam

In memory of author Miguel Huerta.
